# Intelligent Song Recognition via a Hollow‐Microstructure‐Based, Ultrasensitive Artificial Eardrum

**DOI:** 10.1002/advs.202405501

**Published:** 2024-09-20

**Authors:** Shaopeng Li, Jiangtao Tian, Ke Li, Kemeng Xu, Jiaqi Zhang, Tingting Chen, Yang Li, Hongbo Wang, Qiye Wu, Jinchun Xie, Yongjun Men, Weiping Liu, Xiaodan Zhang, Wenhan Cao, Zhongjie Huang

**Affiliations:** ^1^ State Key Laboratory for Modification of Chemical Fibers and Polymer Materials College of Materials Science and Engineering Donghua University Shanghai 201620 China; ^2^ School of Information Science and Technology ShanghaiTech University Shanghai 201210 China; ^3^ School of Electronics and Information Xi'an Polytechnic University Xi'an 710048 China; ^4^ Center for Composites COMAC Shanghai Aircraft Manufacturing Co. Ltd. Shanghai 201620 China

**Keywords:** acoustic sensor, artificial eardrum, hollow microstructure, piezoresistive sensor, song recognition

## Abstract

Artificial ears with intelligence, which can sensitively detect sound—a variant of pressure—and generate consciousness and logical decision‐making abilities, hold great promise to transform life. However, despite the emerging flexible sensors for sound detection, most success is limited to very simple phonemes, such as a couple of letters or words, probably due to the lack of device sensitivity and capability. Herein, the construction of ultrasensitive artificial eardrums enabling intelligent song recognition is reported. This strategy employs novel geometric engineering of sensing units in the soft microstructure array (to significantly reduce effective modulus) along with complex song recognition exploration leveraging machine learning algorithms. Unprecedented pressure sensitivity (6.9 × 10^3^ kPa^−1^) is demonstrated in a sensor with a hollow pyramid architecture with porous slants. The integrated device exhibits unparalleled (exceeding by 1–2 orders of magnitude compared with reported benchmark samples) sound detection sensitivity, and can accurately identify 100% (for training set) and 97.7% (for test set) of a database of the segments from 77 songs varying in language, style, and singer. Overall, the results highlight the outstanding performance of the hollow‐microstructure‐based sensor, indicating its potential applications in human–machine interaction and wearable acoustical technologies.

## Graphical abstract

Artificial eardrums with ultra‐piezoresistive sensitivity are realized via geometrically engineered microstructure units and machine learning for intelligent song recognition. The advanced hollow‐pyramidal‐based sensor achieves unprecedented pressure sensitivity and remarkable sound detection sensitivity, capable of identifying 100% (training data) and 97.7% (test data) of 77 diverse songs, indicating potentials in human–machine interaction and wearable acoustical technologies.

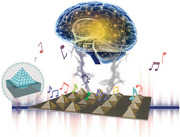

## 1 Introduction

Pressure sensing is of paramount importance to human–machine interaction (HMI),^[^ ^1^ ^]^ healthcare,^[^ ^2^ ^]^ electronic skin,^[^ ^3^ ^]^ tactile technology,^[^ ^4^ ^]^ soundwave detection,^[^ ^5^ ^]^ and Internet of Things.^[^ ^6^ ^]^ Flexible pressure sensors stand out as their flexibility, wearability, lightweight, and adaptability.^[^ ^7^, ^8^, ^9^, ^10^, ^11^ ^]^ Due to the excellent processability and conformability, soft materials have been engineered to soft microstructure arrays (SMAs),^[^ ^12^ ^]^ and such geometry engineering has demonstrated significant advantages in pressure sensing, resulting in high sensitivity, fast response, and improved detection limit.^[^ ^13^, ^14^, ^15^ ^]^ Various unit microstructure patterns, like pillars,^[^ ^16^, ^17^ ^]^ pyramids,^[^ ^18^, ^19^ ^]^ and hemispheres^[^ ^20^, ^21^ ^]^ have been investigated, with pyramid shapes being popular due to their nonuniform stress distribution.^[^ ^22^ ^]^ Though SMA‐based flexible pressure sensors have experienced a great evolution in recent years, most of the investigations are limited to the listed conventional geometries and lacking geometric innovation, with only very few exceptions.^[^ ^12^, ^23^, ^24^ ^]^ It remains obscure that how advanced architecture design of the microstructure unit in a SMA can further manipulate the effective physical property of the material and power the sensing capability of the system for all, or certain forms of pressure.

Sound is a crucial aspect of our perception of life. Among various forms of pressure, sound is a subtle force characterized by a dynamic frequency. Pressure sensors that are highly sensitive in a wide frequency range hold great promise for acoustic sensing,^[^ ^25^, ^26^ ^]^ enabling promising applications as artificial ears in voice biometrics,^[^ ^27^, ^28^ ^]^ voice‐controlled HMI,^[^ ^29^ ^]^ and wearable healthcare devices.^[^ ^30^ ^]^ For example, sensitive flexible resistive acoustic/vibration sensors, which have been attached to a throat or placed in front of a loudspeaker,^[^ ^31^, ^32^, ^33^, ^34^, ^35^, ^36^, ^37^ ^]^ can convert analog acoustic waves/vibrations into electric data (resistance changes),^[^ ^38^, ^39^, ^40^ ^]^ which are often further processed using machine learning (ML) algorithms to reduce noise and enhance interpretation.^[^ ^35^, ^41^, ^42^ ^]^ Learning from human cognition, these ML algorithms efficiently decipher inherent data patterns, substantiating a requirement for comprehensive understanding of both soft acoustic/vibration sensors and their corresponding ML algorithms for maximizing the benefits of these sensors in complex application contexts. However, despite the emerging investigation of flexible sensors for sound and voice detection, most success has been limited to very simple phonemes, such as a couple of letters or words^[^ ^35^, ^40^, ^41^, ^42^, ^43^, ^44^, ^45^, ^46^, ^47^ ^]^ (as summarized in Table S1 in the Supporting Information), probably due to the lack of device sensitivity and limited exploration of ML algorithms. Therefore, there is a critical need in such a system for the recognition of more complex sounds in real‐life scenarios, such as songs, which encompass lyrics, melodies, and rhythms, and pose greater challenges for recognition.

Intelligent artificial ears hold great promise in revolutionizing our lifestyle. In this work, we showcase an elegant strategy to build an intelligent artificial eardrum, based on the breakthrough in geometric engineering of the sensing unit for ultrasensitive pressure sensing, and novel exploration of complex song recognition with ML algorithms. Guided by the principle to reduce the effective modulus of the sensing unit, we designed a series of novel hollow pyramidal SMAs and demonstrated unprecedented piezoresistive sensitivity (6.9 × 10^3^ kPa^−1^) in the sensor with hollow pyramids and porous slant architecture. The device successfully functioned as an intelligent artificial eardrum for ultrasensitive sound detection, and compared with reported flexible sensors, our sensor shows extraordinary sensitivity (exceeding by 1–2 orders of magnitude). Further, our artificial eardrum was able to recognize songs with a high accuracy of 100% and 97.7% in a database of the segments of 77 songs for training and test data, respectively. To the best of our knowledge, this is the first reported example of complex song recognition with a flexible sensor which is cheap and easy to fabricate. These results underscore the superb performance of our hollow‐pyramidal‐SMA‐based pressure sensor and suggest huge potential of its application in HMI and wearable electronics.

## 2 Results and Discussion

**Figure 1:**
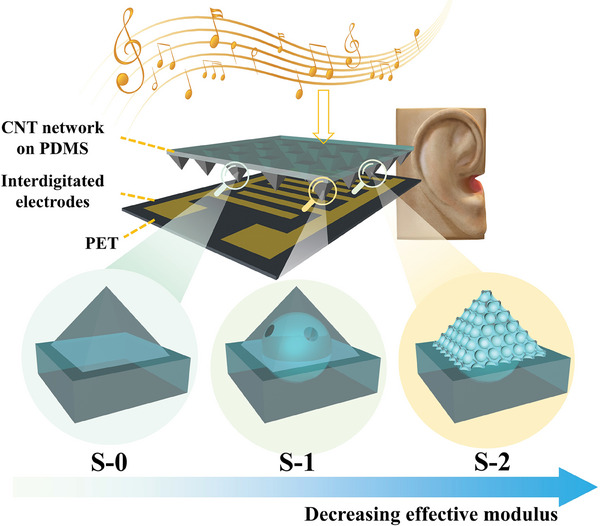
Design principle of an artificial eardrum based on a piezoresistive senor with a microstructure array with innovative unit architectures. A series of pyramidal microstructures were designed and prepared: with S‐0 referring to conventional solid one, S‐1 the hollow structure with a spherical hole, and S‐2 the advanced hollow structure with porous pyramid slants.

Our fundamental design principle is to reduce the effective modulus of the sensing unit in the SMA through architecture innovation, to test the direct correlation between unit compressibility and sensor sensitivity. As the central building block of our piezoresistive sensors for potential application as artificial eardrums, a series of distinct pyramidal SMAs were designed and fabricated with the help of a premachined template with pyramidal holes. The detailed preparation protocols can be found in the Experimental Section. As illustrated in **Figure** **1**, S‐0 represents the conventional solid pyramidal array, which can be easily fabricated using the Si template,^[^ ^48^ ^]^ and is often used in sensing,^[^ ^49^ ^]^ electrodes,^[^ ^50^ ^]^ and soft lithography.^[^ ^51^, ^52^ ^]^ S‐1 and S‐2 are novel generations of SMAs that we designed with hollow structure. As illustrated in Figure S1 (Supporting Information), we developed innovative protocols to fabricate S‐1 and S‐2, utilizing air bubbles and uniform‐sized polystyrene (PS) microspheres in the preparation process, respectively. While S‐1 successfully realized the hollow pyramidal structure, S‐2 adopted further complexity for the hierarchical architecture, by introducing porous slants for all the pyramids in an array.

**Figure 2:**
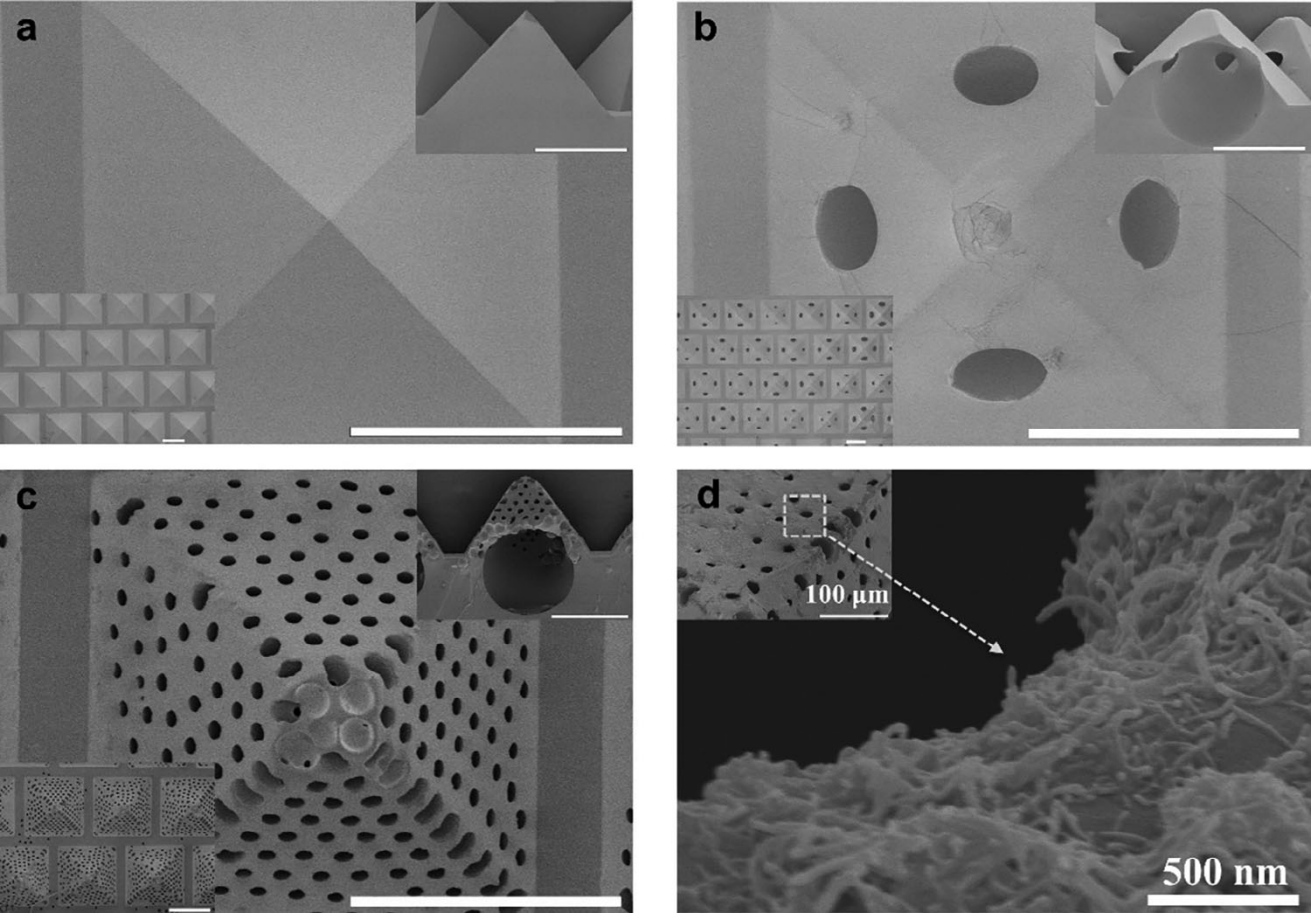
S‐0‐, S‐1‐, and S‐2‐based SMAs. SEM image of a) S‐0, b) S‐1, and c) S‐2, with the bottom left inset showing an organized pyramidal array, and the top right inset indicating the cross‐section of structure. d) S‐2 with a surface‐coated CNT conductive layer. All scale bars represent 300 µm unless otherwise noted.

The prepared S‐0, S‐1, and S‐2 geometries were distinct and consistent over the whole array. S‐0 represents an array of conventional solid pyramids (as shown by the scanning electron microscopy (SEM) image in **Figure** **2**a) with a base length of 500 µm. The inset indicates the uniform size and orderly arrangement of each pyramid structure. In addition, S‐1 (see Figure 2b) kept a spherical hole (with a radius of ≈205 µm, as can be seen in the top right inset) inside each pyramid, making for the hollow pyramid structure. Furthermore, enabled by the self‐assembly of PS microspheres (see Figure S2 in the Supporting Information) on the surface of those pyramidal holes of the template and postetching with toluene, S‐2 slants of the pyramids were filled with small holes left by the etched PS microspheres (see Figure 2c), each with a radius of ≈15 µm and a pitch of around 50 µm. Similar to S‐1, S‐2 was equipped with a large spherical hole within each pyramid, as illustrated by the cross‐section image. For the spherical holes in S‐1 and S‐2, while top hemispheres lay inside the pyramids, the bottom hemispheres were imbedded in the polydimethylsiloxane (PDMS) backing layer (see the optical transmittance image in Figure S3 in the Supporting Information). It should be noted that we managed to control the position of the spherical hole via the setup of the heating procedure (see Figure S4 in the Supporting Information). Therefore, herein we provide a universal and fundamental approach to generate advanced hollow hierarchical microstructures by manipulating the bubble in the elastomer curing process, with good controllability of the position and size of the central spherical hole, and the approach is compatible with the Si template method which has been widely used for SMA fabrication.^[^ ^48^ ^]^

The fabricated pyramidal SMAs were then spay‐coated with conductive carbon nanotube (CNT) inks (with an average CNT length of 0.7 µm). The conductive networks were formed on the surface of SMAs, as characterized by Figure 2d and Figure S5 (Supporting Information). The surface‐conductive SMAs were coupled with flexible interdigitated electrodes to form flexible piezoresistive pressure sensors. The sensing principle is due to the resistance change of the surface CNT conductive network upon pressure, which is caused by the deformation of the microstructure unit.^[^ ^20^, ^35^, ^53^, ^54^, ^55^, ^56^, ^57^, ^58^, ^59^ ^]^

**Figure 3:**
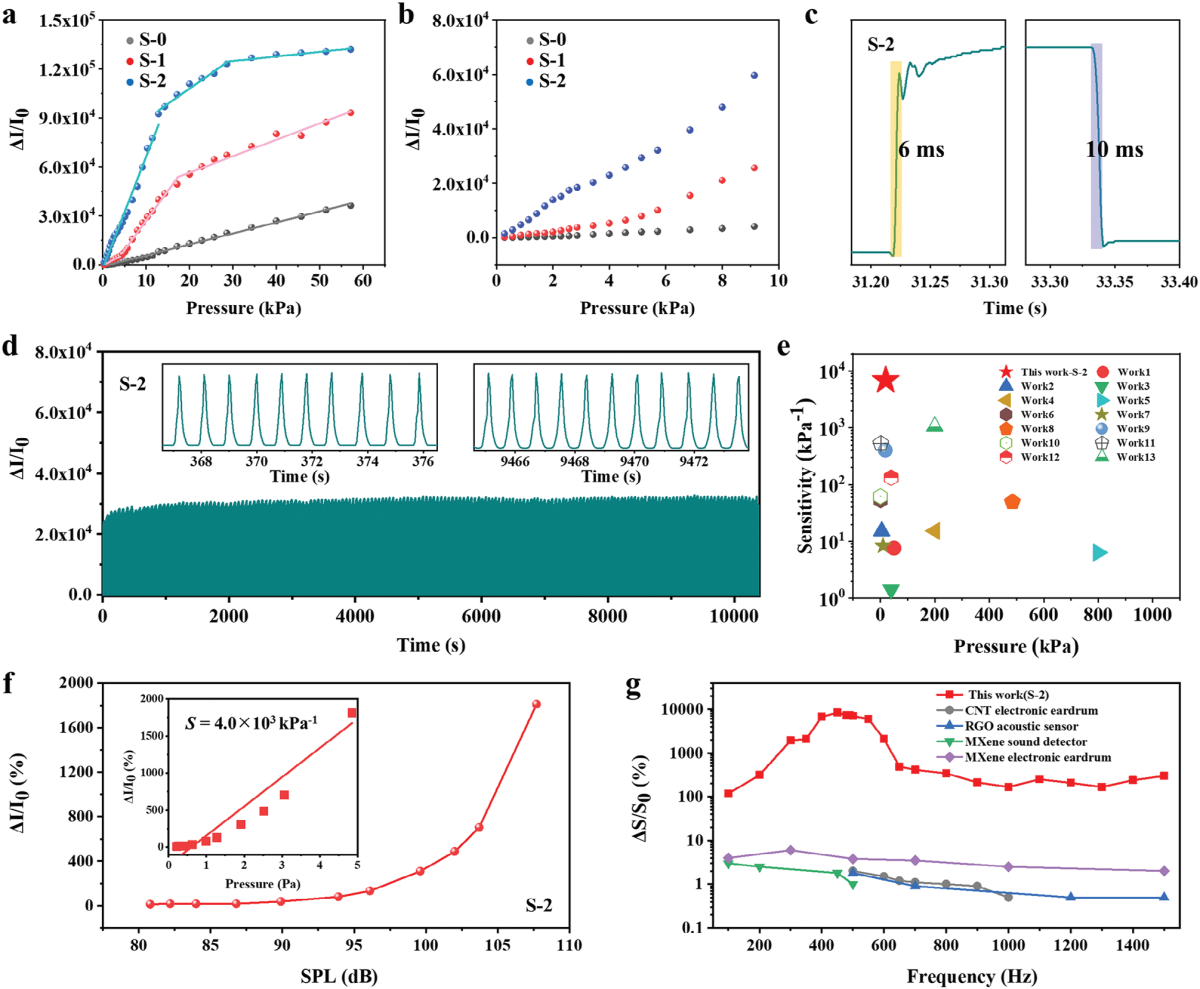
Pressure and acoustic sensing performance of sensors S‐0, S‐1, and S‐2. Comparison of sensitivity curves of S‐0, S‐1, and S‐2 in a) the full range of 0–60 kPa and b) a zoom in at low pressure region. c) Time‐dependent current plot of S‐2 showing the response and recovery processes. d) Cycling test of S‐2 for 12 000 cycles at a pressure load of 5 kPa. e) Systematic comparison of the sensitivity of S‐2 with other recently reported microstructure‐based flexible pressure sensors (with details summarized in Table S2 in the Supporting Information). S‐2 serves as an ultrasensitive acoustic sensor, as demonstrated by f) the significant dependence of signal response on sound pressure level (SPL) at 300 Hz (the inset highlights the estimated sensitivity corresponding to sound pressure change), and g) a comparison showing the signal response (Δ*S*/*S*_0_) of S‐2 is comprehensively better than other reported flexible sensors across a wide frequency range of 100–1500 Hz.

The performance of the piezoresistive pressure sensor was demonstrated to depend on the geometry engineering of the SMA unit. The sensing performance of the device based on S‐0, S‐1, and S‐2 unit structures (noted as sensor S‐0, S‐1, and S‐2) was systematically evaluated by measuring the relative current change under a constant voltage responding to various applied compression forces. **Figure** **3**a outlines the performance of sensor S‐0, S‐1, and S‐2, with sensitivity *S* represented in the figure as the slope of the curve. Sensor S‐0, which lacks additional hierarchical structures, demonstrated a sensitivity of 6.8 × 10^2^ kPa^−1^ throughout the entire pressure testing range. Sensor S‐1 and S‐2 clearly exhibit much higher sensitivity in the overall 0–60 kPa range. In fact, the characteristic values (4.0 × 10^3^ kPa^−1^ at the pressure range of 4.5–14.3 kPa for S‐1 and 6.9 × 10^3^ kPa^−1^ at the pressure range of 0–14 kPa for S‐2) reach about 6 and 10.5 times the sensitivity of S‐0. Though the sensitivity of S‐2 decreased to 2.8 × 10^2^ kPa^−1^ within the pressure range of 28–60 kPa, which can be due to the completely compressed structure at high‐pressure regime, it cannot overshadow the crucial discovery that S‐2 is ultrasensitive in low pressure range, as clearly highlighted in Figure 3b. It is noteworthy that both sensors S‐1 and S‐2 displayed multiple linear regions in Figure 3a, a trend commonly observed in many reported flexible pressure sensors.^[^ ^20^, ^53^, ^54^, ^56^, ^57^, ^58^, ^59^, ^60^ ^]^ This behavior is attributed to the response of varying components within the hierarchical microstructures.

All the three sensors showed the excellent Ohmic contact, as can be validated from the *I*–*V* curves under various applied pressures, which display ideal linear *I*–*V* relationships and an increase in slope (indicating a decrease in resistance) commensurate with an increase in compression force (see Figure S6a–c in the Supporting Information). They are also adept at distinguishing various levels of force (see Figure S6d–f in the Supporting Information). Further, sensor S‐2 exhibited an impressive response and recovery times of 6 and 10 ms, respectively (refer to Figure 3c).

Considering cyclic stability as another key metric to assess the capability of pressure sensors, sensor S‐2 was subjected to as many as 12 000 cycles of testing under 5 kPa of loading pressure (Figure 3d). Each cycle was completed in less than 1 s, with Δ*I*/*I*_0_ remaining highly constant. The illustration of the first and last 10 cycles (indicated by the *I*–*t* curve), which were almost identical and displayed ≈106% sensitivity retention, proves the exceptional cyclability of the device. This test unambiguously demonstrated that the delicate S‐2 microstructure is ultradurable for deformation cycling. We also investigated the effect of the size of the conductive unit (i.e., CNT) in this system. When longer CNTs (with an average length of 7 µm) were utilized, the sensitivity of S‐2 slightly dropped (see Figure S7 in the Supporting Information). Therefore, the conductive network formed by the short CNTs is more likely to induce resistance change, probably due to more heterogeneous interface between different tubes.

The comparison of sensitivity and detection range of the pressure sensors has been shown in Figure 3e, adopting the format widely presented in the literature.^[^ ^35^, ^61^, ^62^, ^63^, ^64^ ^]^ S‐2 not only outperformed S‐0 and S‐1 in our work, but also demonstrated unprecedented sensitivity compared to other recently reported SMA‐based counterparts in this field (detailed in Table S2 in the Supporting Information) in the low‐to‐middle pressure range.^[^ ^20^, ^21^, ^35^, ^53^, ^59^, ^65^, ^66^, ^67^, ^68^, ^69^, ^70^, ^71^, ^72^ ^]^ Thus, it is suitable for the sensitive detection of subtle mechanical force or sound, e.g., acoustic sensing. Therefore, we successfully demonstrated that ultrasensitive piezoresistive sensing can be achieved through the geometric engineering of the SMA units: the spherical holes inside the pyramid and the porous slants dramatically reduce the effective compression modulus of the microstructure, thereby increasing the sensitivity of the sensor.

As an ultrasensitive flexible pressure sensor, S‐2 was tested for acoustic sensing and demonstrated its superiority among the field. Variations in the current as a function of sound pressure level (SPL) at a frequency of 300 Hz are illustrated in Figure 3f. Observation reveals that the sensor S‐2′s current response bears a positive correlation with the SPL, exhibiting a significant value as high as 1800% at 107 dB. The sound pressure sensitivity of our sensor is remarkably estimated at ≈4.0 × 10^3^ kPa^−1^ (as shown in the inset). This impressive performance stands out when compared to the pioneering work of Gou et al.^[^ ^35^ ^]^ who, using a similar setup for an artificial eardrum, reported a sound detection sensitivity of 62 kPa^−1^. Our sensor achieves a sensitivity that is one to two orders of magnitude higher than the benchmark set by the literature, underscoring a leap forward in sound detection technology. The signal response (Δ*S*/*S*_0_) of different acoustic detectors at varied frequencies is depicted in Figure 3g. The systematic comparison shows that the response of S‐2 is comprehensively better than other reported flexible sensors across a wide frequency range of 100–1500 Hz. Furthermore, sensors S‐0, S‐1, and S‐2 all exhibited remarkable sensitivity improvements at frequencies below 700 Hz, with peak performances around 300–500 Hz (see Figure S8 in the Supporting Information), a characteristic commonly viewed in resistive sensors.

**Figure 4:**
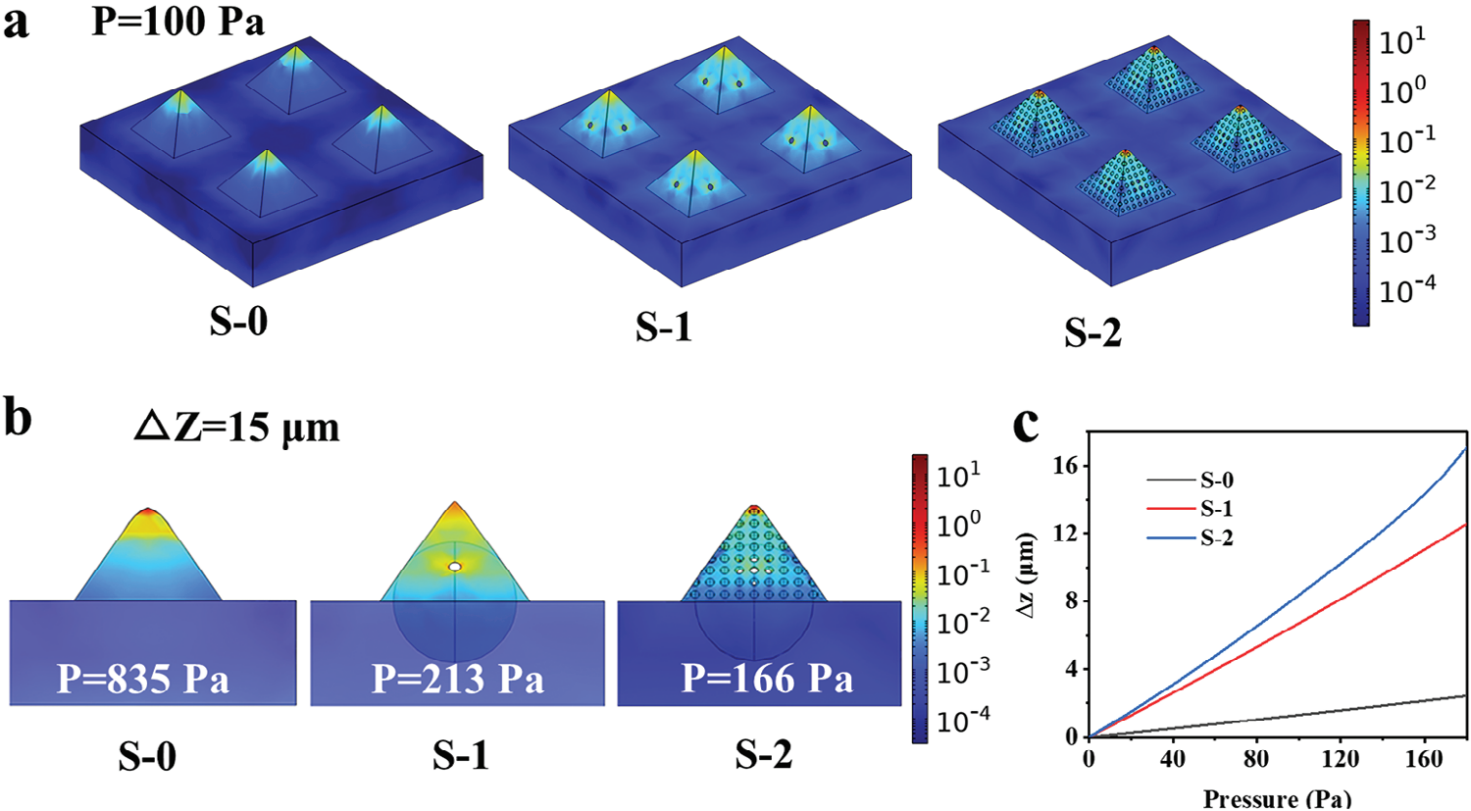
Sensing mechanism of the sensors (S‐0, S‐1, and S‐2) simulated by FEA. a) Stress distribution plots of S‐0, S‐1, and S‐2 under the same external pressure (100 Pa), showing that the more complex the structure, the greater the stress concentration effect (S‐0 < S‐1 < S‐2). The unit of the scale bar is MPa. b) Stress distribution plots of S‐0, S‐1, and S‐2 with the same longitudinal deformation, showing that the more complex the structure, the smaller the force required (S‐0 > S‐1 > S‐2). The unit of the scale bar is MPa. c) Longitudinal deformation–loading force curves for the three sensors.

The underlying pressure‐response mechanism of the series of microstructures (S‐0, S‐1, and S‐2) was investigated by finite element analysis (FEA) simulation. The FEA simulation results revealed distinct stress distributions and deformation characteristics for the three pyramidal microstructures under varying external pressure loads. The stress distribution diagrams (**Figure** **4**a) illustrate how the complexity of the microstructure influenced the stress patterns, with hollower structures exhibiting greater stress concentration effect under the same external pressure. Figure 4b shows the magnitude of the external pressure loads to be applied and the corresponding stress distribution for the three structures with an identical longitudinal deformation (Δ*z*) of 15 µm. As the structure becomes hollower, the external load required to produce the same degree of deformation is less. The longitudinal deformation variation curve depicted in Figure 4c indicates that the deformation response of the microstructures to the applied external pressure load is highly sensitive. Among them, S‐2 exhibits the highest sensitivity, while the S‐0 structure shows the lowest. We find that the simulation results agree reasonably well with the experimental discoveries.

The extraordinary pressure sensing performance of sensor S‐2 was meticulously evaluated for its potential in real‐life applications as wearable technology, demonstrating its sensitivity and versatility. As illustrated in Figure S9a (Supporting Information), the sensor exhibited significant current variations even in response to the subtle impact of a freely dropped leaf, underscoring its high sensitivity. When integrated into a common office mouse, the sensor produced distinct current changes with standard clicking actions (see Figure S9b in the Supporting Information), highlighting its capability to accurately monitor operational dynamics in everyday devices. Further showcasing its practical value, sensor S‐2 consistently delivered clear and repetitive signal outputs from the wrist pulses of a 24 years old male volunteer, measured at the radial artery (see Figure S9c in the Supporting Information). This enabled precise heart rate calculation and evaluation, with the inset graph detailing the different stages of a normal pulse wave. Additionally, when positioned on the wrist, the sensor effectively detected wrist flexion angles, indicating a direct correlation between increasing electrical current and greater wrist bending angles (see Figure S9d in the Supporting Information). Expanding its applications, the sensor S‐2 demonstrated remarkable sensitivity when attached to the throat, reacting to vibrations caused by speaking and swallowing. During the vocalization of “hello, everyone” in Mandarin, the sensor distinctly identified pressure variations resulting from subtle muscular movements, producing unique current peaks corresponding to each syllable (see Figure S9e in the Supporting Information). Moreover, the sensor's response to swallowing was marked by characteristic peaks (see Figure S9f in the Supporting Information), further emphasizing its high sensitivity and reproducibility. These findings collectively reveal sensor S‐2 as a highly promising candidate for various wearable technology applications, encompassing health monitoring, human–computer interaction, and beyond. The sensor's capability to translate minute physical interactions into precise electrical signals paves the way for responsive and adaptive wearable devices.

Sensor S‐2 was tested as an artificial eardrum for voice sensing and recognition. We recorded the time‐dependent variations in S‐2 signal waveforms generated by a loudspeaker. The phrase “good morning” said by a 28 years old female volunteer was recorded by an iPhone and played through the loudspeaker, and the resulting signals of the S‐2 sensor were recorded. The temporal response of the S‐2 closely mirrored the audio signals recorded by the iPhone, as evidenced in Figure S10a (Supporting Information). The reasonably good synchronization between the S‐2 sensor's waveform and the iPhone's audio waveform demonstrates S‐2′s acoustic sensing capability, indicating comparable performance with commercial voice recorders. Similarly, this kind of phrase recognition was used in a voice security authentication model utilizing the S‐2 sensor. Our approach involved recording the phrase 11 times spoken by two volunteers (one male and one female) under various conditions. These recordings were then played through a speaker, with the S‐2 sensor capturing the signal. The 22 voice data samples obtained were used as the training set. Additionally, the two volunteers each provided an extra recording which served as the test set. After 30 iterations, our model achieved an outstanding 99.4% accuracy in distinguishing the unique voice waveforms and spectrums of volunteers A and B. The high level of precision indicates the feasibility of designing a voiceprint lock, granting security access only to specific individuals who speak the authorized phrase. Figure S10b (Supporting Information) showcases the iteration curves of both the training and test sets using our proposed convolutional neural network (CNN) model. As depicted, accuracy improves with the number of iterations. The training set achieved an average recognition accuracy of 99.8%, while the test set attained an average accuracy of 99.4%, demonstrating the impressive classification performance of our model. This paves the way for enhanced voice‐based security systems.

**Figure 5:**
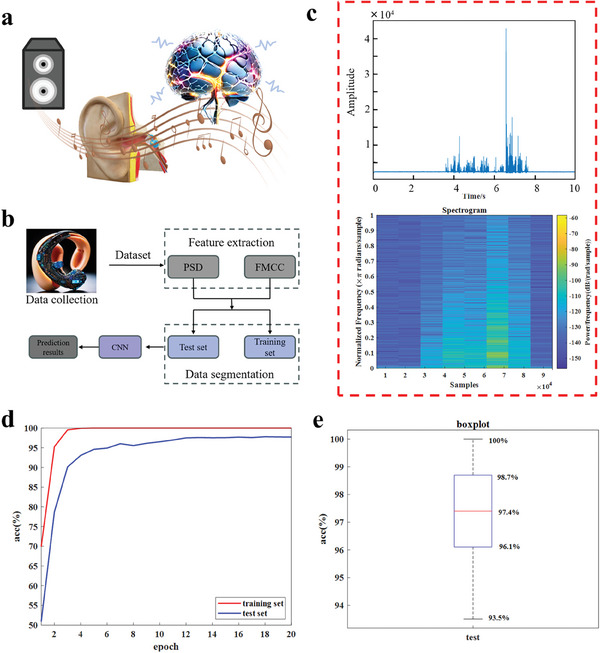
Sensor S‐2 for application as intelligent artificial eardrum. a) Schematic of an artificial eardrum for sound recognition. b) The ML recognition flow diagram. c) An example of (top) the recorded electrical signal of a segment of a Mandarin pop song “Mermaid” (by JJ Lin) played on and off in the middle of a 10 s recording, and (bottom) its acoustic spectrum obtained by short‐time Fourier transform. d) Iteration curves for training and test sets. e) Boxplot of the test set.

The exploration of intelligent song recognition, with the help of ML algorithms, was displayed in **Figure** **5**. The song recognition task (as illustrated in Figure 5a) has not been reported for flexible acoustic sensors, to the best of our knowledge, probably due to the complexity and difficulty of the task itself. Song recognition tends to pose greater challenges than conventional limited‐word recognition, because songs usually comprise various acoustic elements, including vocals, accompaniment, and harmony. This complexity makes it incredibly tough to isolate lyrics or melodic lines from background sounds, which significantly complicates song recognition and categorization.

As illustrated in the ML processing flow (Figure 5b), the power spectral density (PSD) and mel frequency cepstrum coefficient (MFCC) of each vibration signal were extracted as the feature set, and the dataset was divided into training and test sets, and then they were sent to CNN for recognition. Based on this process, the segments of 77 songs with varying artists, languages, and music styles were first played by the loudspeaker and documented through the S‐2 artificial eardrum with a sampling rate of 10 000 s^−1^. A typical time‐dependent current signal of a recording with a song turned on and off in the middle of the recording was shown as the top of Figure 5c, and short‐time Fourier transform was utilized to examine the recorded signals, with the resultant spectrogram shown as the bottom of Figure 5c. It can be clearly seen that the signal fluctuation in the time and frequency domains is consistent, confirming the sensor's outstanding frequency response. The signals of each voice recorded per same segment of the song were nearly identical (as demonstrated in Figure S11 in the Supporting Information), validating the device's commendable stability and repeatability. Within the ML framework, 77 data sets were designated as training set, and another round of data recorded with the 77 segments of the songs played again were allocated as testing set. As the number of training epochs increased, the accuracy of both the training and test sets showed an upward trend (see Figure 5d). Before the 4th epoch, both showed a significant upward trend, and the accuracy of the training set had reached 100% on 4th epoch, the test set reached 97.7% on 20th epoch. As shown in Figure 5e, the highest accuracy of test set was 100% with the median of 97.4% (with a standard deviation of 0.0171) on 20th epoch, proving the outstanding accuracy.

Confusion matrices, which illustrate the discrepancy between the samples’ prediction and its true classification, imply high accuracy for each sample and reflect the device's consistent stability (as depicted in Figure S12 in the Supporting Information). The features extracted in the paper can effectively represent the speech information of songs, and CNN can also adapt to the features, achieving good classification results. Consequently, our ML‐algorithm‐based intelligent artificial eardrum demonstrated enormous potential for HMI.

## 3 Conclusion

In summary, we have successfully demonstrated that we can tune the sensitivity of flexible piezoresistive sensors via the geometric engineering of the microstructure unit. We found that the microstructure of hollow pyramids with porous slants enables ultrasensitive pressure sensing and sound detection, as corroborated by both experimental and simulated results. We report an artificial eardrum with superior sensitivity compared to existing literature. We also effectively employed a machine learning model for the processing of data collected by this artificial eardrum. Accuracy rates of 100% and 97.7% were achievable for training and test datasets, respectively, for recognizing songs of distinct languages, styles, and artists. These findings suggest that our artificial eardrum possesses huge promise for HMI and wearable acoustical devices.

## 4 Experimental Section

### Fabrication of SMAs with S‐0, S‐1, and S‐2 Designs

SMAs with designed microstructures were fabricated on a modified template approach, as outlined in Figure S1 (Supporting Information). S‐0 was fabricated using the premachined Si template, while the PDMS template with pyramidal holes was used to fabricate S‐1 and S‐2. For the preparation of S‐1, a doctor‐blading method was employed to evenly spread a layer of liquid PDMS (Sylgard 184 prepolymer and cross‐linker with a mass ratio of 10:1)^[^ ^73^ ^]^ on the template before immediate thermal cross‐linking at 100 °C for 1 h. An instant heating on the 100 °C hot plate was necessary to ensure that the formed spherical bubbles did not overflow, and the control example is shown in Figure S4 (Supporting Information).

For the preparation of S‐2, a PS ethanolic dispersion was first obtained via solvent exchange. The solvent of PS aqueous dispersion (2.5 wt%, with a particle size of 40 µm, purchased from Rigor Science Co., Ltd.) was replaced by ethanol, through centrifuge, solvent exchange, and ultrasonication for multiple cycles. The PS ethanolic dispersion was deposited on the surface of the PDMS template, allowing self‐assembly of PS microspheres. After the natural evaporation of ethanol, a layer of orderly packed PS microspheres formed on the template surface. Excess PS microspheres on the surface were blown away with an ear wash bulb. Then, the procedure in S‐1 preparation was adopted. After thermal curing, the resulted SMA was immersed in a toluene solution for ultrasonication for 3 h, yielding S‐2 after the complete etching of PS microspheres.

### Pressure Sensing Tests

CNT networks were used as the conductive component for the device.^[^ ^74^ ^]^ The SMAs were uniformly spray‐coated with a lab‐made CNT–*m*‐cresol ink (CNT: XFNANO Materials Tech Co.).^[^ ^75^, ^76^ ^]^ As characterized by SEM for over 35 samples on Si wafer, the average length of CNT was determined as 0.7 µm. The long CNTs (purchased from Timesnano, with a characterized average length of 7 µm) were also adopted in the work for a control study. The surface‐conductive SMAs were assembled with a Au‐interdigitated electrode for pressure sensing. Various pressures were exerted onto the sensors utilizing a universal tensile testing machine (UTM2103, Shenzhen Suns Technology Stock Co., LTD.), and current data collection was executed with an electrochemical workstation (Reference 620, Gamry). The test was conducted at a constant applied voltage of 0.5 V. Sensitivity (*S*) was calculated as *S* = Δ(Δ*I*/*I*_0_)/Δ*P*, where *P* represents pressure applied to the sensor, Δ*I* is the current change under differing pressure *P*, and *I*_0_ is the initial current when there was no pressure.

### FEA Simulations

A series of FEA simulations were carried out utilizing COMSOL Multiphysics 6.0 software. The base model consisted of a 2 × 2 array of pyramidal microstructures. Each microstructure had a base length of 500 µm, a height of 353 µm, and a bottom substrate thickness of 353 µm. The detailed parameter settings for structures S‐1 and S‐2 were consistent with the experimental findings. The material was identified as PDMS with a Young's modulus of 1.1 MPa. In order to apply a stabilizing external force from the top of the pyramid downward, an additional structural layer was placed at the apex of the model. This layer had the same geometry as the bottom substrate and was made of rigid metal. The bottom of the bottom substrate was defined as a fixed boundary, while a boundary load (*P*) was applied to the upper surface of the top metal structure. The external load *P* used to simulate the three microstructures (S‐0, S‐1, and S‐2) ranged from 0 to 2000, 700, and 180 Pa, respectively. The variation in the simulation range of the load *P* was partially related to the model complexity and meshing difficulty. By adjusting the value of *P*, the mechanical behavior and deformation characteristics of the pyramidal microstructure were simulated under various pressure conditions.

### Acoustic Sensing Tests

Sensor S‐2 was affixed on the surface of a compact loudspeaker (Xiaomi Communications Co., Ltd.). While subjected to an environmental noise level of 80 dB, the loudspeaker played a range of vocals along with discrete sound signals and songs from different artists, languages, and music styles. The SPL of the sound was accurately measured by a sound level meter. The electrochemical workstation (Reference 620, Gamry) was used to collect the changes in the sensor's electrical current resulting from the sound waves, attaining a sampling rate of 10 000 s^−1^. The conversion from dB to sound pressure was based on the previously reported equation.^[^ ^35^ ^]^ A systematic protocol was adopted to test the signal response of S‐0, S‐1, and S‐2 in the frequency range of 100–1500 Hz. Employing the mobile application “sound frequency generator” (developed by Sichuan Banya Network Technology Co., Ltd.), an audio signal with fixed (decided by cellphone input) frequency was played continuously for 5 s (at around 100–110 dB for all frequencies). The electrochemical workstation collected the sensors’ electrical signal under the constant applied bias of 0.5 V, enabling the evaluation of the sensors’ signal response (change in current) under the specific acoustic frequency.

For song recognition, segments of overall 77 songs from diverse artists (e.g., Queen, G.E.M., JJ Lin, Beyond) featuring various languages (e.g., English, Madeiran, Cantonese) were played in the middle of those 10 s recordings. During data processing, PSD and MFCC were extracted as the characteristic features. Standardizing the extracted feature data was then proceeded to adhere to a normal distribution. Subsequently, a CNN model was selected in constructing the classification model. Regarding the selection of parameters for CNN, this study adopted a learning rate of 0.0005 and a batch size of 8. Additionally, the training set was simultaneously employed as a validation set to enhance the recognition capability of the model.

## Conflict of Interest

The authors declare no conflict of interest.

## Supporting information



Supporting Information

## Data Availability

The data that support the findings of this study are available from the corresponding author upon reasonable request.
