# Supplementary material for: Intelligent Song Recognition via a Hollow‐Microstructure‐Based, Ultrasensitive Artificial Eardrum
Source: Adv Sci (Weinh). 2024 Sep 20;11(42):2405501. doi: 10.1002/advs.202405501 (PMC11558140; doi:10.1002/advs.202405501)
Supplement: Supplementary file 1 — Supporting Information [file ADVS-11-2405501-s001.pdf]

ADVANCED SCIENCE
Open Access

Supporting Information

for *Adv. Sci.*, DOI 10.1002/advs.202405501

Intelligent Song Recognition via a Hollow-Microstructure-Based, Ultrasensitive Artificial Eardrum

Shaopeng Li, Jiangtao Tian, Ke Li, Kemeng Xu, Jiaqi Zhang, Tingting Chen, Yang Li, Hongbo Wang, Qiye Wu, Jinchun Xie, Yongjun Men, Weiping Liu, Xiaodan Zhang, Wenhan Cao* and Zhongjie Huang**

Supporting Information

Intelligent Song Recognition via a Hollow Microstructure-Based, Ultrasensitive Artificial Eardrum

Shaopeng Li, Jiangtao Tian, Ke Li, Kemeng Xu, Jiaqi Zhang, Tingting Chen, Yang Li, Hongbo Wang, Qiye Wu, Jinchun Xie, Yongjun Men, Weiping Liu, Xiaodan Zhang, Wenhan Cao,* and Zhongjie Huang**

Table S1. Summary of the recent progress of flexible acoustic sensors for voice and speech detection and recognition. Most success were limited to a few letters and words.

Materials	Sound type	Location	Accuracy	Year	Ref.
MXene/PDMS	Seven words	Loudspeaker	95%	2022	[1]
$Cu_3(HHTP)_2$ MOF	Instrumental classical music without lyrics	Loudspeaker	61%	2020	[2]
Molybdenum Disulfide/ Hydroxyethyl Cellulose/Polyurethane Sponge	Seven words	Throat	97.14%	2022	[3]
MXene/bacterial cellulose	Natural sounds; Poetry recitation	Loudspeaker	-	2022	[4]
MXene/hydrogel	Four letters	Throat	95%	2023	[5]
Silver-coated nanofibers	Four letters	Ear	92.64%	2022	[6]
Silver nanowire	One word	Throat	99.1%	2018	[7]
Polytetrafluoroethylene /Polyethylene terephthalate	Five letters	Loudspeaker	97.33%	2021	[8]
$Nd_2Fe_{14}B$/PDMS	Four short phrases	Throat	99%	2020	[9]

Table S2. Summary of the recent progress of microstructured flexible pressure sensors.

Materials	Shape	Sensitivities(kPa^{-1})	Measuring range	Year	Work
Graphene/PU foam	Porous	7.62 (0-50 kPa)<br>0.14 (50-200 kPa)	0-200 kPa	2020	Work 1[10]
Graphene/PDMS	Porous	15.22 (0-5 kPa)<br>0.51 (5-45 kPa)	0-45 kPa	2020	Work 2[11]
rGO	Porous	0.82 (10-25 kPa)<br>1.43 (25-40 kPa)	0-40 kPa	2020	Work 3[12]
rGO/PDMS	Hemispherical microstructures	15.4	0-200 kPa	2020	Work 4[13]
Graphite flakes/PDMS	Micropillar	6.4	0-800 kPa	2020	Work 5[14]
(Polyaniline/ polyvinylidene fluoride)/Au/PDMS	Microdome	53	58.4-960 Pa	2021	Work 6[15]
Carbon black/PDMS	Hemispherical at different height levels	8.3 (0-10 kPa)<br>4.3 (10-30 kPa)<br>1.5 (30-200 kPa)	0-200 kPa	2021	Work 7[16]
Poly(vinyl alcohol)/ H_3PO_4	Hemispheres with fine pillars	49.1	0-485 kPa	2022	Work 8[17]
MXene/PDMS	Convex structure	164.93 (0-10 kPa)<br>403.46 (10-18 kPa)	0-18 kPa	2022	Work 9[18]
MXene/PDMS	Pyramid	62	0-1.25 Pa	2022	Work 10[1]
MXene/bacterial cellulose/PDMS	Microcone	65.41 (0-0.6 kPa)<br>528.87 (0.6-2 kPa)<br>10.45 (2-10 kPa)	0-10 kPa	2023	Work 11[19]
rGO/PDMS	Submicrostructures	133.003 (0-40 kPa)<br>11.369 (100-300)	0-300 kPa	2023	Work 12[20]
rGO/PDMS	Multilevel microstructured	2.5 (0.01-1 kPa)<br>12.0 (1-50 kPa)<br>1051 (50-200 kPa)<br>470 (200-400 kPa)	0-400 kPa	2019	Work 13[21]

Figure S1. Illustration of fabrication protocols of SMAs with S-0, S-1 and S-2 design.

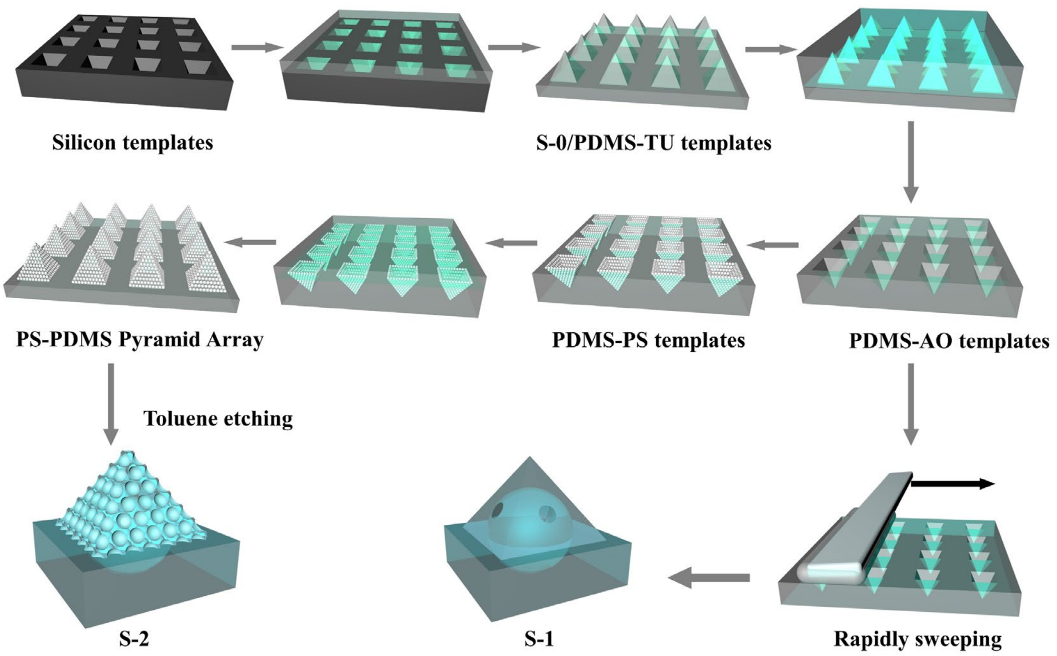

Figure S2. SEM images showing the self-assembly of PS microspheres on the surface of a pyramidal hole of the template.

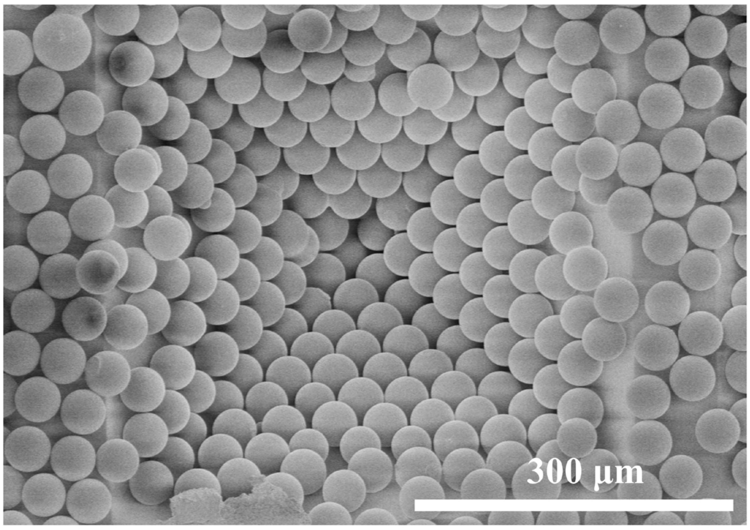

Figure S3. Optical microscope images of (a) S-0, (b) S-1, and (c) S-2, showing well-defined spherical holes inside both S-1 and S-2's pyramids.

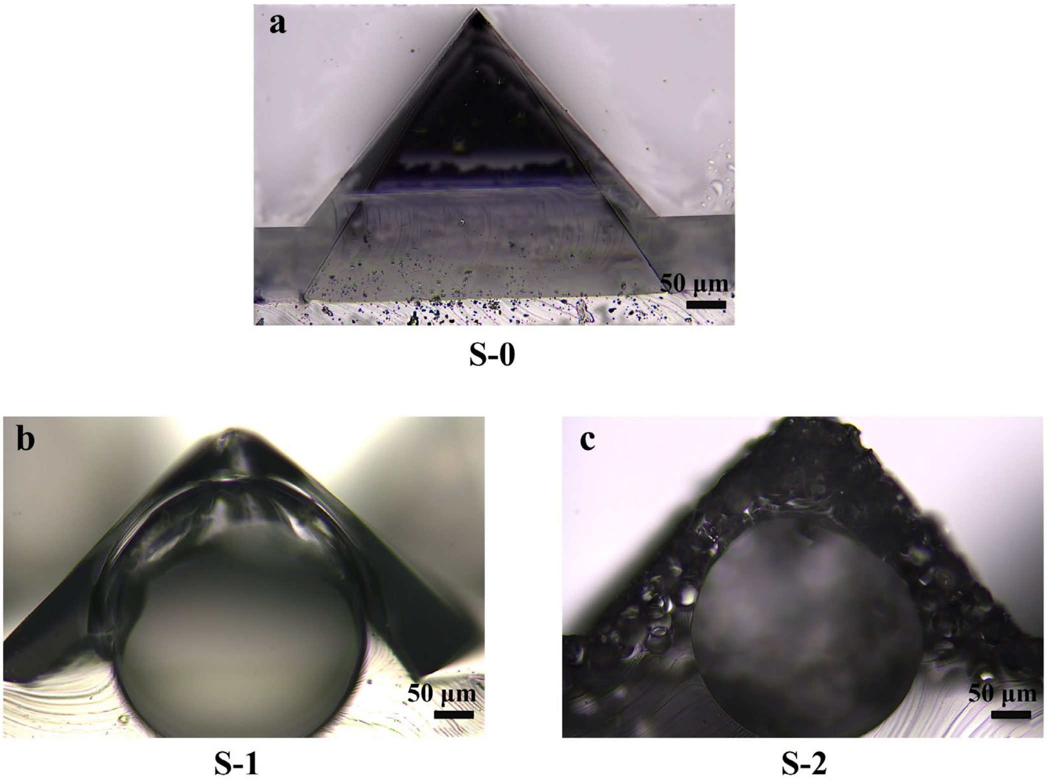

Figure S4. Optical microscope images illustrating the position of the spherical holes inside the pyramid, fabricated by heating on a 100°C-hot plate with a specific rest time after the doctor-blading process: (a) 60 s, (b) 5 s. The result demonstrates that an instant heating on the hot plate is crucial for maintaining the position of the spherical holes. The scale bar represents 400 μm.

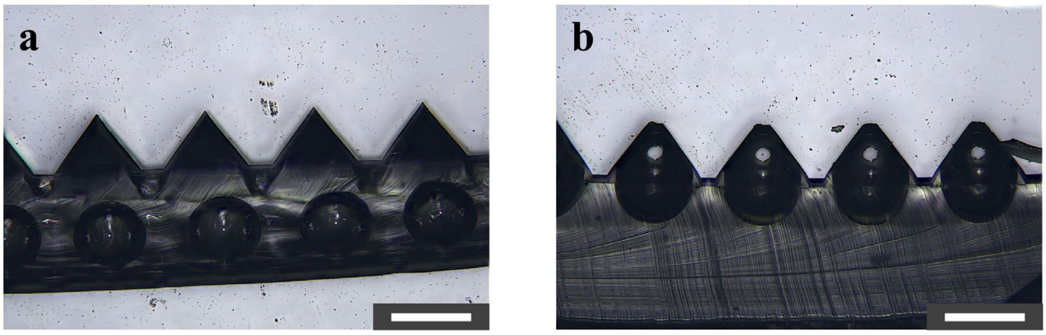

Figure S5. SEM images showing the CNT conductive layer uniformly coated on the SMA surfaces: (a) S-0, (b) S-1, and (c) S-2. The scale bar represents 400 μm.

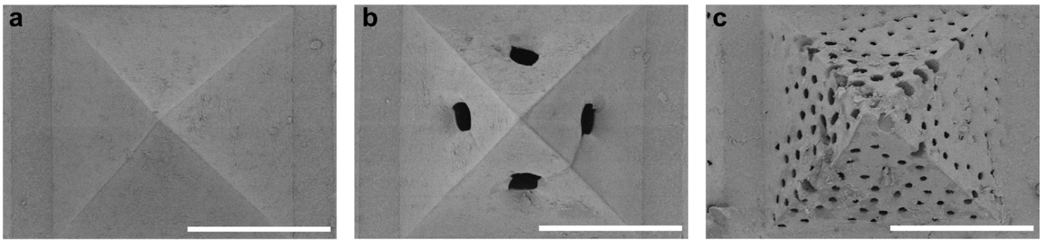

Figure S6. Sensing performance tests of sensor S-0, S-1, and S-2. (a)-(c) show the *I-V* plots of the sensors, all showing excellent ohmic contact. (d)-(f) show the time-dependent current variation plots of the sensors at different pressures, demonstrating excellent adaptability and stability.

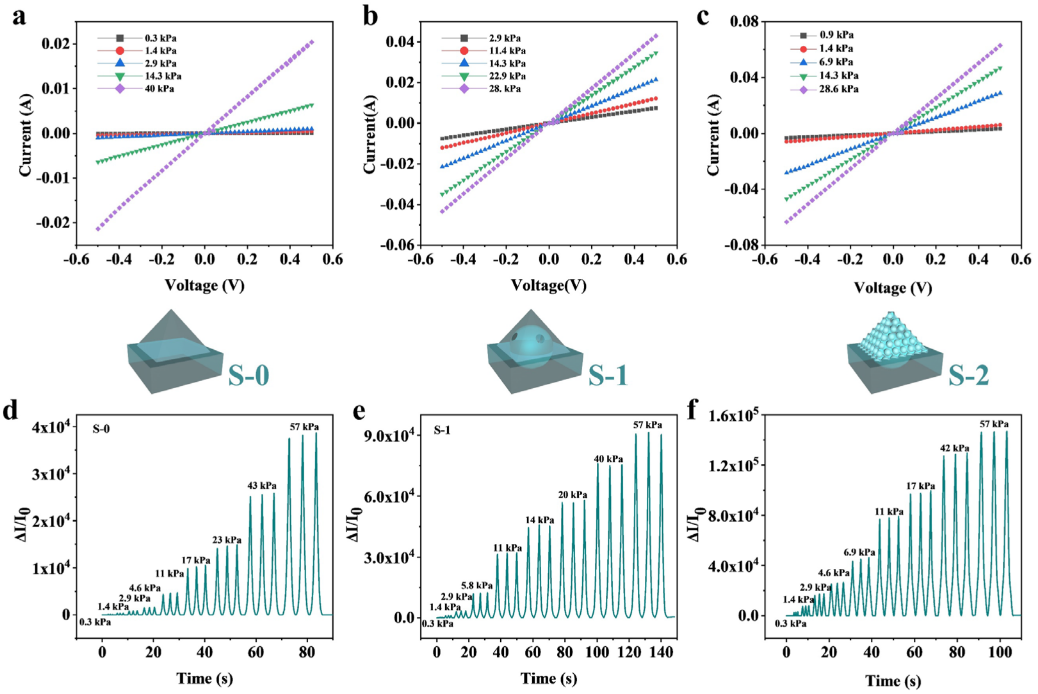

Figure S7. Long CNT-based S-2 sensor shows a declined sensitivity compared with that of the short CNT-based one.

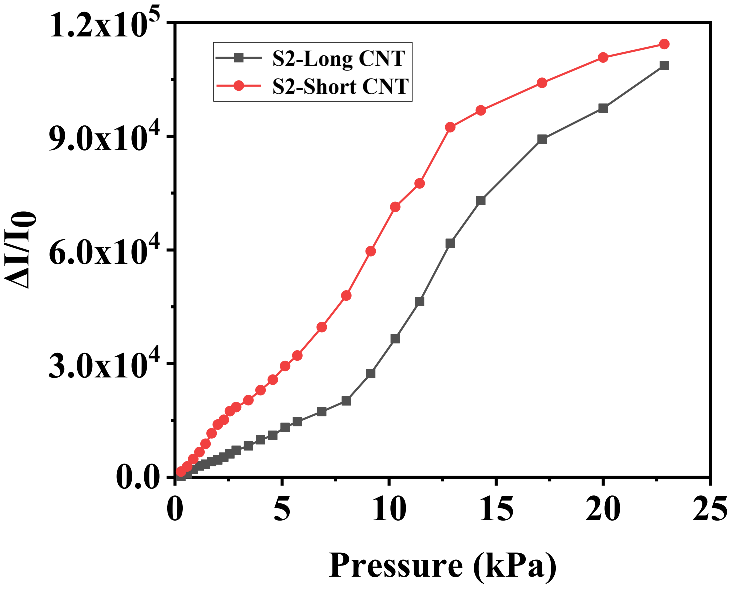

Figure S8. Signal sensitivity of S-0, S-1, and S-2 with response to various frequencies at 100-1500 Hz regime.

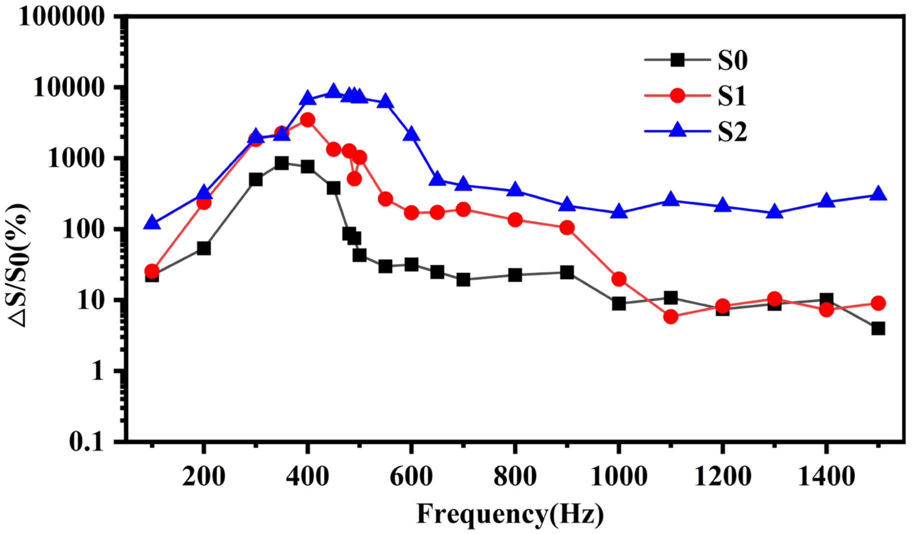

Figure S9. Real-life applications of S-2 as a wearable sensing device for: (a) a falling leaf; (b) computer mouse clicking; (c) pulse at the wrist, the inset shows an enlarged waveform of a pulse vibration; (d) bending the wrist at different angles; (e) the vibration of the throat when the volunteer says “Hello Everyone” (in Mandarin, “Da Jia Hao”); and (h) the vibration of the throat as the volunteer performs a swallowing action.

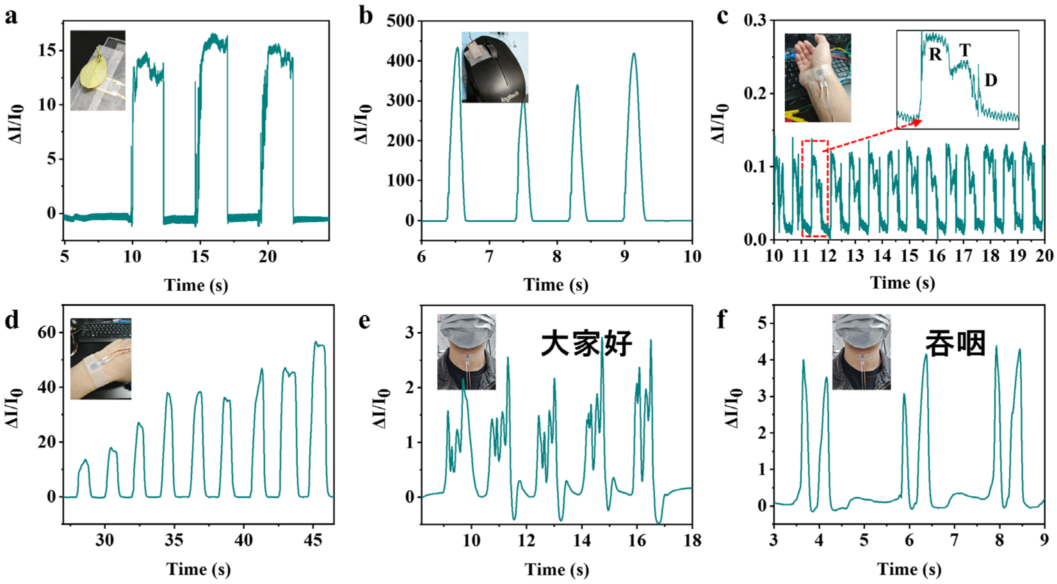

Figure S10. S-2 was tested for voice sensing and recognition. (a) We recorded the time-dependent variations in S-2 signal waveforms generated by a loudspeaker. The phrase "good morning" said by a 28-year-old female volunteer was recorded by an iPhone and played through the loudspeaker, and the resulting signals of the S-2 sensor were recorded. (b) The ML iteration curves of both the training and test sets using our CNN model for a voiceprint lock system. The training set achieved an average recognition accuracy of 99.8% while the test set attained an average accuracy of 99.4%.

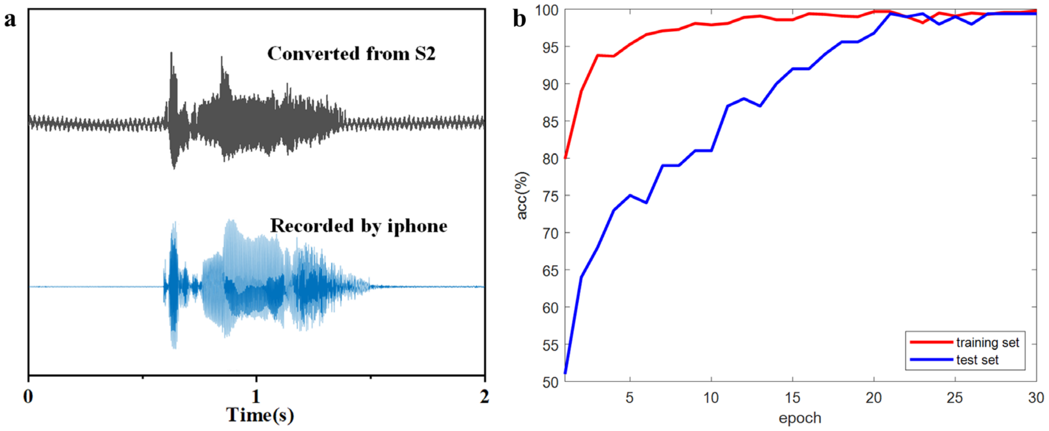

Figure S11. The signals of two recordings of the same segment of the song (“River South”, a famous Mandarin pop song by JJ Lin), showing highly repeatable signal patterns.

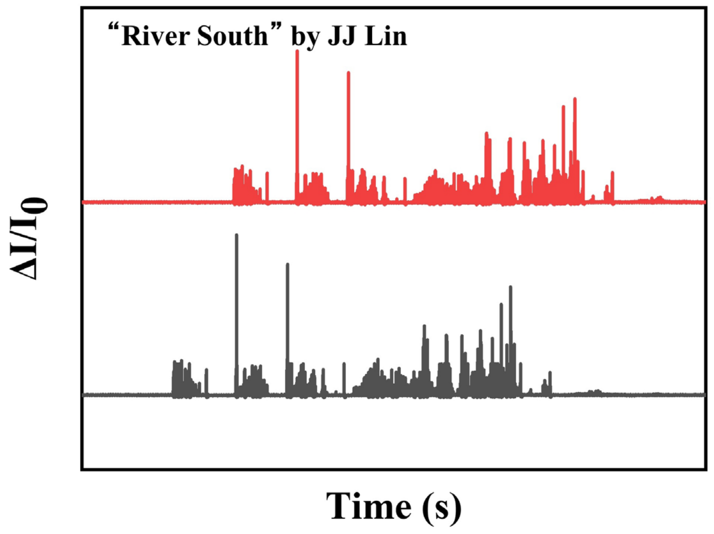

Figure S12. Confusion matrix of test set of overall 77 songs.

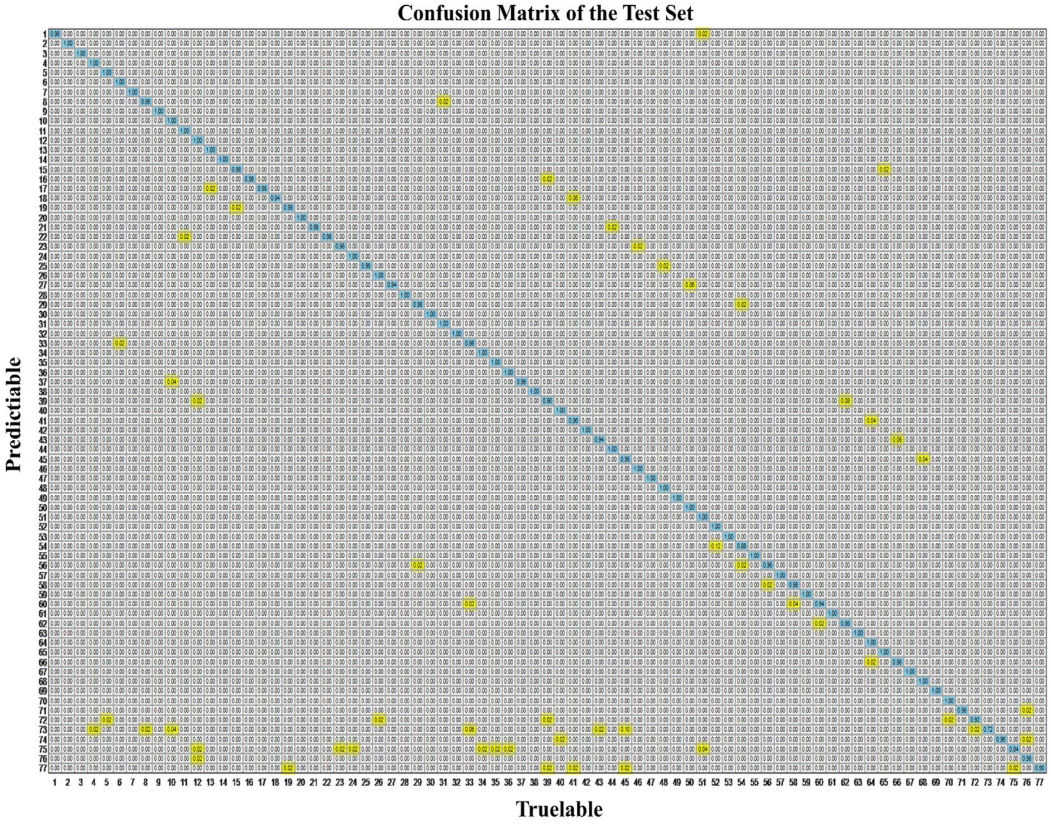

Supplementary References

(1) Gou, G.-Y.; Li, X.-S.; Jian, J.-M.; Tian, H.; Wu, F.; Ren, J.; Geng, X.-S.; Xu, J.-D.; Qiao, Y.-C.; Yan, Z.-Y.; et al. Two-stage amplification of an ultrasensitive MXene-based intelligent artificial eardrum. *Sci. Adv.* **2022** 8 (13), 2156.
(2) Zhou, K.; Zhang, C.; Xiong, Z.; Chen, H.-Y.; Li, T.; Ding, G.; Yang, B.; Liao, Q.; Zhou, Y.; Han, S.-T. Template-directed growth of hierarchical MOF hybrid arrays for tactile sensor. *Adv. Funct. Mater.* **2020**, 30 (38), 2001296.
(3) Chen, X.; Zhang, D.; Luan, H.; Yang, C.; Yan, W.; Liu, W. Flexible pressure sensors based on molybdenum disulfide/hydroxyethyl cellulose/polyurethane sponge for motion detection and speech recognition using machine learning. *ACS Appl. Mater. Interfaces* **2023**, 15 (1), 2043.
(4) Su, T.; Liu, N.; Lei, D.; Wang, L.; Ren, Z.; Zhang, Q.; Su, J.; Zhang, Z.; Gao, Y. Flexible MXene/bacterial cellulose film sound detector based on piezoresistive sensing mechanism. *ACS Nano* **2022**, 16 (5), 8461.
(5) Chen, J.; Xia, X.; Yan, X.; Wang, W.; Yang, X.; Pang, J.; Qiu, R.; Wu, S. Machine learning-enhanced biomass pressure sensor with embedded wrinkle structures created by surface buckling. *ACS Appl. Mater. Interfaces* **2023**, 15 (39), 46440.
(6) Jiang, Y.; Zhang, Y.; Ning, C.; Ji, Q.; Peng, X.; Dong, K.; Wang, Z. L. Ultrathin eardrum-inspired self-powered acoustic sensor for vocal synchronization recognition with the assistance of machine learning. *Small* **2022**, 18 (13), 2106960.
(7) Kang, S.; Cho, S.; Shanker, R.; Lee, H.; Park, J.; Um, D.-S.; Lee, Y.; Ko, H. Transparent and conductive nanomembranes with orthogonal silver nanowire arrays for skin-attachable loudspeakers and microphones. *Sci. adv.***2018**, 4 (8), 8772.
(8) Lin, Z.; Zhang, G.; Xiao, X.; Au, C.; Zhou, Y.; Sun, C.; Zhou, Z.; Yan, R.; Fan, E.; Si, S.; et al. A personalized acoustic interface for wearable human–machine interaction. *Adv. Funct. Mater.* **2022**, 32 (9), 2109430.
(9) Zhao, Y.; Gao, S.; Zhang, X.; Huo, W.; Xu, H.; Chen, C.; Li, J.; Xu, K.; Huang, X. Fully flexible electromagnetic vibration sensors with annular field confinement origami magnetic membranes. *Adv. Funct. Mater.* **2020**, 30 (25), 2001553.
(10) Feng, C.; Yi, Z.; Jin, X.; Seraji, S. M.; Dong, Y.; Kong, L.; Salim, N. Solvent crystallization-induced porous polyurethane/graphene composite foams for pressure sensing. *Composites Part B-Eng.* **2020**, 194, 108065.
(11) Wang, Y.; Wu, H.; Xu, L.; Zhang, H.; Yang, Y.; Wang, Z. L. Hierarchically patterned self-powered sensors for multifunctional tactile sensing. *Sci. adv.* **2020,** 6 (34), 9083.
(12) Chai, Y.; Ma, X.; Zang, X.; Wang, X.; Zhu, N.; Xie, Z.; Ji, J.; Zhang, X.; Xue, M. Cold direct pen writing of reduced graphene oxide foams for ultrasensitive micro-contact force probing. *Carbon* **2020**, 157, 140.
(13) Li, Z.; Zhang, B.; Li, K.; Zhang, T.; Yang, X. A wide linearity range and high sensitivity flexible pressure sensor with hierarchical microstructures via laser marking. *J. Mater. Chem. C* **2020**, 8 (9), 3088.
(14) Xu, H.; Gao, L.; Wang, Y.; Cao, K.; Hu, X.; Wang, L.; Mu, M.; Liu, M.; Zhang, H.; Wang, W.; et al. Flexible waterproof piezoresistive pressure sensors with wide linear working range based on conductive fabrics. *Nanomicro Lett.* **2020**, 12 (1), 159.
(15) Yang, T.; Deng, W.; Chu, X.; Wang, X.; Hu, Y.; Fan, X.; Song, J.; Gao, Y.; Zhang, B.; Tian, G.; et al. Hierarchically microstructure-bioinspired flexible piezoresistive bioelectronics. *ACS Nano* **2021**, 15 (7), 11555.
(16) Geng, D.; Chen, S.; Chen, R.; You, Y.; Xiao, C.; Bai, C.; Luo, T.; Zhou, W. Tunable wide range and high sensitivity flexible pressure sensors with ordered multilevel microstructures. *Adv. Mater. Technol.* **2022**, 7 (6), 2101031.

(17) Bai, N.; Wang, L.; Xue, Y.; Wang, Y.; Hou, X.; Li, G.; Zhang, Y.; Cai, M.; Zhao, L.; Guan, F.; et al. Graded interlocks for iontronic pressure sensors with high sensitivity and high linearity over a broad range. *ACS Nano* **2022**, 16 (3), 4338.
(18) Yan, J.; Ma, Y.; Jia, G.; Zhao, S.; Yue, Y.; Cheng, F.; Zhang, C.; Cao, M.; Xiong, Y.; Shen, P.; et al. Bionic MXene based hybrid film design for an ultrasensitive piezoresistive pressure sensor. *Chem. Eng. J.* **2022**, 431, 133458.
(19) Yang, J.; Liu, L.; Zhang, D.; Zhang, H.; Ma, J.; Zheng, J.; Wang, C. Dual-stage surficial microstructure to enhance the sensitivity of MXene pressure sensors for human physiological signal acquisition. *ACS Appl. Mater. Interfaces* **2024**, 16 (1), 1096.
(20) Zhang, Y.; Wang, Y.; Wang, C.; Zhao, Y.; Jing, W.; Wang, S.; Zhang, Y.; Xu, X.; Zhang, F.; Yu, K.; et al. Superior performances via designed multiple embossments within interfaces for flexible pressure sensors. *Chem. Eng. J.* **2023**, 454, 139990.
(21) Tang, X.; Wu, C.; Gan, L.; Zhang, T.; Zhou, T.; Huang, J.; Wang, H.; Xie, C.; Zeng, D. Multilevel microstructured flexible pressure sensors with ultrahigh sensitivity and ultrawide pressure range for versatile electronic skins. *Small* **2019**, 15 (10), 1804559.
